# Clinical pregnancy outcomes in young women with diminished ovarian reserve undergoing frozen embryo transfer: a comprehensive analysis with exploratory insights into endometrial aging

**DOI:** 10.3389/fendo.2025.1608200

**Published:** 2025-07-29

**Authors:** Feng-xia Liu, Hui-xin Ming, Ka-li Huang, Shan-jia Yi, Xue-fei Liang, Wei-wei Luo, Ming-hua Shi

**Affiliations:** ^1^ Department of Reproductive Medicine, Reproductive Hospital of Guangxi Zhuang Autonomous Region, Nanning, Guangxi, China; ^2^ Department of Pathology, The People`s Hospital of Guangxi Zhuang Autonomous Region, Nanning, Guangxi, China

**Keywords:** diminished ovarian reserve, endometrial factors, exploratory analysis, p16, pregnancy outcomes

## Abstract

**Introduction:**

This study compared pregnancy outcomes after frozen-thawed embryo transfer (FET) in infertile women aged <40 years with diminished ovarian reserve (DOR) versus normal ovarian reserve (NOR), incorporating exploratory analysis of potential endometrial aging factors.

**Methods:**

In this retrospective study, we analyzed the data of 1,362 patients aged <40 years who underwent FET between January and December 2024. Patients were categorized into two groups: the DOR (anti-Müllerian hormone [AMH] < 1.1 ng/mL, n = 136) and NOR (AMH ≥ 1.1 ng/mL, n = 1,226) groups. Pregnancy outcomes were compared after adjusting for confounding factors using inverse probability weighting. Additionally, exploratory immunohistochemical analysis of p16 expression was performed using endometrial samples from 16 patients (n = 8 per group).

**Results:**

After weighting, the clinical pregnancy rate was significantly lower in the DOR group than in the NOR group (47.0% vs. 58.3%, P = 0.040; odds ratio = 0.63, 95% confidence interval: 0.41–0.98). Exploratory analysis revealed that the expression of p16 was significantly higher in the endometrial cells of patients with DOR than in those of patients in the NOR group (P < 0.001). Furthermore, a trend toward lower clinical pregnancy rates was observed with higher p16 expression.

**Conclusion:**

These exploratory findings suggest that reduced pregnancy rates in young women with DOR may involve endometrial aging mechanisms; however, the preliminary nature and limited sample size for molecular analysis necessitate cautious interpretation and warrant validation in larger and well-controlled cohorts.

## 1 Introduction

Diminished ovarian reserve (DOR) refers to a decrease in the quantity and quality of the oocytes within the ovaries and is clinically characterized by low levels of the anti-Müllerian hormone (AMH), antral follicle count, and elevated basal follicle-stimulating hormone levels (1). The essence of DOR is an ovarian functional decline, which can be age-related or caused by other factors, leading to premature ovarian aging. DOR affects approximately 20% of the infertile population, with an increasing trend in younger individuals (2, 3). This decline in ovarian reserve is associated with fewer oocytes and compromised developmental potential, which complicates natural conception and often necessitates reliance on assisted reproductive technology such as *in vitro* fertilization (IVF)/intracytoplasmic sperm injection (ICSI)-embryo transfer and frozen-thawed embryo transfer (FET). However, patients with DOR face specific challenges in assisted reproductive technology, including limited oocyte yield, fewer high-quality embryos, and increased cycle cancellation rates, ultimately resulting in significantly lower pregnancy rates than those of women with normal ovarian reserve (4). Improving pregnancy outcomes in patients with DOR is both a major challenge and a focal point in the field of assisted reproduction.

The effect of DOR on pregnancy rates in younger women remains controversial, with studies using various age cutoffs ranging from 35 to 40 years. In older patients with DOR (>38 years), age-related ovarian decline has a pronounced effect on oocyte quantity and quality, adversely affecting *in vitro* fertilization/intracytoplasmic sperm injection outcomes (5). Some studies report that DOR lowers clinical pregnancy and live birth rates in younger women undergoing fresh embryo transfer cycles (6), whereas others suggest that pregnancy rates are similar between women with DOR and age-matched women with normal ovarian reserves when embryos are transferred (7). These conflicting findings suggest that the reproductive outcomes in young patients with DOR may be influenced by factors other than ovarian function.

Successful implantation requires an adequate high-quality embryo and receptive endometrium (8). While the focus in patients with DOR has traditionally been on the limited number and compromised quality of oocytes, the potential impact of endometrial factors on implantation success has received relatively less attention. Patients with DOR exhibit a tendency toward ovarian aging, which may also be accompanied by endometrial aging. A diminished ovarian function may also induce systemic alterations in the reproductive axis, potentially influencing the uterine microenvironment (9). Emerging evidence links cellular aging to impaired endometrial receptivity (10). Studies have shown stable implantation, pregnancy, and birth rates in women aged 25–40 years who underwent egg donation, whereas fertility declines in older recipients. Compared to younger donors who use their own oocytes for embryo transfer, older recipients exhibit decreased endometrial receptivity, suggesting that endometrial aging is a significant factor affecting pregnancy success (11).

Endometrial aging may impair receptivity through cellular senescence, inflammatory responses, structural and functional changes, epigenetic modifications, extracellular matrix alterations, and impaired angiogenesis (12). At the cellular level, aging is characterized by irreversible cell cycle arrest, telomere shortening, DNA damage accumulation, and release of senescence-associated secretory phenotype (SASP) factors, all of which can hinder embryo-uterine interactions (13). A key molecular marker of cellular aging is a single marker (p16 INK4a, cyclin-dependent kinase inhibitor 2A, CDKN2A), which plays a role in cell cycle arrest, senescence maintenance, and oxidative stress regulation (14). Notably, p16 expression was significantly upregulated in the endometrial epithelium of older women, indicating that cellular senescence, characterized by markers such as p16INK4a, has been implicated in age-related decline in endometrial receptivity (12). Nevertheless, the potential contribution of endometrial aging to impaired receptivity in DOR patients remains underexplored and limited in the current literature. Further studies are needed to investigate whether premature endometrial aging occurs in young DOR patients and contributes to reduced pregnancy success.

This study primarily aimed to investigate clinical pregnancy outcomes in young patients with DOR following FET, with particular attention to potential endometrial factors. Accordingly, in this exploratory study, we examined patients under 40 years of age with DOR to test the primary hypothesis that young patients with DOR exhibit lower clinical pregnancy rates following FET compared to those with normal ovarian reserve (NOR). We defined young women as those <40 years of age, consistent with recent literature recognizing that reproductive aging effects become more pronounced after the age of 40 years (15, 16). As a secondary exploratory objective, we investigated whether endometrial p16 expression is upregulated in a subset of DOR patients, providing preliminary evidence for potential endometrial aging as a contributing mechanism.

## 2 Materials and methods

This study was approved by the ethics committee of reproductive hospital of Guangxi Zhuang autonomous region (No: KY-LW-2025-06). We designed this study as an exploratory investigation to identify potential associations between DOR, endometrial aging, and pregnancy outcomes, with the aim of providing preliminary data for hypothesis generation and guiding subsequent large-scale mechanistic studies. This study comprises two components: (i) a primary clinical outcome analysis examining pregnancy rates after FET in 1,362 young women (<40 years old) with DOR and (i) an exploratory molecular investigation of endometrial aging markers in a subset of 16 patients to generate preliminary evidence for mechanistic hypotheses.

### 2.1 Participants

We retrospectively analyzed the clinical data of patients who underwent FET at Guangxi Zhuang Autonomous Region Reproductive Hospital between January 2024 and December 2024. The inclusion criteria were as follows: age <40 years, first embryo transfer cycle following initial oocyte retrieval, and availability of complete clinical data. The exclusion criteria were as follows: the presence of hydrosalpinx, adenomyosis, endometriosis, uterine abnormalities (e.g., septate uterus, unicornuate uterus, and bicornuate uterus), intrauterine adhesions, or endometrial pathologies (e.g., endometrial hyperplasia, chronic endometritis, and endometrial polyps). Additionally, patients with abnormal endometrial ultrasound findings before transfer, such as heterogeneous echotexture or endometrial separation, were excluded.

The included patients were categorized into two groups based on the ovarian reserve status: the DOR (AMH < 1.1 ng/mL) and NOR (AMH ≥ 1.1 ng/mL) groups (17). Among them, 16 patients (8 from each group) who underwent hysteroscopy and endometrial curettage during the proliferative phase were randomly selected. Paraffin-embedded endometrial tissue samples from the curettage procedures were retrieved for immunohistochemical (IHC) analysis.

### 2.2 Treatment protocol

Protocols for fresh IVF/ICSI stimulation cycles were selected based on individual patient factors in accordance with the 2019 ESHRE guidelines on ovarian stimulation (18).

The initial gonadotropin dosage was determined based on individual parameters and adjusted according to ovarian response. When more than three follicles reached a diameter ≥18 mm, triggering was performed using either Chorionic Gonadotrophin 2000 IU + Recombinant Human Choriogonadotropin alfa 250 µg or a gonadotropin-releasing hormone agonist (GnRH-a) 0.2 mg + Chorionic Gonadotrophin 2000 IU. Oocyte retrieval was conducted 34–38 h after triggering under transvaginal ultrasound guidance. The retrieved oocytes were fertilized via intracytoplasmic sperm injection or conventional IVF and cultured to the cleavage or blastocyst stage. Embryos that met these criteria were cryopreserved for future use. Good-quality cleavage-stage embryos were defined as grades I and II, with 7–11 cells. Good-quality blastocysts were defined as blastocysts of grade 3BB or higher.

Endometrial preparation for FET was performed during the natural cycle, ovulation induction cycle, hormone replacement therapy (HRT) cycle, or GnRH-HRT cycle, according to the patient’s specific condition and menstrual cycle characteristics.

1. Natural cycle: The transvaginal ultrasound was performed starting on Day 10 of the menstrual cycle to monitor follicular development and endometrial thickness until ovulation, with the day of ovulation designated as Day 0 (D0). Luteal phase support begins on D0 using either progesterone capsules (150 mg, twice daily), dydrogesterone (20 mg, twice daily), or progesterone injections (40 mg, once daily). Embryo transfer was scheduled on Day 3 for cleavage-stage embryos and Day 5 for blastocysts.
2. Ovulation induction cycle: Between Days 2 and 5 of the menstrual cycle, transvaginal ultrasound and urinary human chorionic gonadotropin (hCG) tests were performed to exclude pregnancy. Ovulation was initiated using tamoxifen and human menopausal gonadotropin (HMG). Follicular development and endometrial thickness were monitored using transvaginal ultrasonography until ovulation, with the day of ovulation designated D0. Luteal phase support started on D0 with either progesterone capsules (150 mg, twice daily), dydrogesterone (20 mg, twice daily), or progesterone injections (40 mg, once daily). Embryo transfer was conducted on Day 3 for cleavage-stage embryos and on Day 5 for blastocysts.
3. HRT cycle: Between Days 2 and 5 of the menstrual cycle, transvaginal ultrasound and urinary hCG tests were performed to rule out pregnancy. If the endometrial thickness is <6 mm, estradiol valerate is initiated at 3 mg once daily, with regular monitoring to adjust the dosage until the endometrial thickness reaches ≥8 mm. Once the target endometrial thickness was achieved, progesterone supplementation was initiated using either soft progesterone capsules (200 mg, twice daily) or dydrogesterone (20 mg, twice daily), with the first day of progesterone administration designated as D0. Embryo transfer was then performed on Day 3 for cleavage-stage embryos or on Day 5 for blastocysts.
4. GnRH-HRT cycle: Between Days 2 and 5 of the menstrual cycle, transvaginal ultrasound and urinary hCG tests were performed to rule out pregnancy, followed by the administration of a GnRH agonist (3.75 mg) for downregulation. After 28 days, estradiol valerate (3 mg once daily) was initiated with regular monitoring of endometrial thickness, and the dosage was adjusted until the endometrial thickness reached ≥8mm. Progesterone supplementation was initiated using either soft progesterone capsules (200 mg, twice daily) or dydrogesterone (20 mg, twice daily), with the first day of progesterone administration designated as D0. Embryo transfer was subsequently performed on Day 3 for cleavage-stage embryos and on day 5 for blastocysts.

### 2.3 Outcome measures

The primary outcomes were hCG positivity, clinical pregnancy, and biochemical pregnancy loss. hCG positivity was defined as a serum hCG ≥25 mIU/mL at 14 days after FET. Clinical pregnancy was defined as the presence of an intrauterine gestational sac with detectable fetal cardiac activity on transvaginal ultrasonography performed 28 days after FET. Biochemical pregnancy loss was defined as a positive hCG test, but no gestational sac or evidence of ongoing pregnancy was observed by ultrasound 28 days after embryo transfer. These standardized timepoints were applied to all patients regardless of the transferred embryo stage (both cleavage-stage embryos and blastocysts), consistent with our center’s unified assessment protocol.

### 2.4 IHC staining, imaging acquisition, and analysis

At our center, hysteroscopy is routinely performed as part of the standard pre-FET workup to evaluate the uterine cavity and exclude intrauterine abnormalities that could affect implantation. The hysteroscopy is performed during the proliferative phase of the menstrual cycle preceding the FET cycle. All patients included in the IHC subanalysis had normal hysteroscopic findings, confirming the absence of intrauterine pathology that could confound endometrial aging assessment.

Endometrial tissues were dehydrated, embedded in paraffin, and sectioned into 3-mm-thick slices for subsequent IHC staining and analysis. Paraffin sections were processed using the Roche Ventana automated IHC staining system with a primary antibody specific to p16 (anti-p16 INK4A antibody, clone: mx007, ready-to-use). Finally, sections were mounted and prepared for further analysis. Both positive and negative control groups were included in the experiment. After washing, samples were incubated with an HRP-labeled secondary antibody at room temperature. DAB was used as the chromogen for staining, followed by hematoxylin counterstaining. The sections were then dehydrated and mounted for further analysis.

IHC-stained slides were analyzed under an upright microscope equipped with a photo-capture system. Appropriate fields were selected under low magnification (×4), and typical images were captured under high magnification (×40). Five random fields of view were analyzed per slide. The positive cell rate was quantified using ImageJ software by calculating the proportion of p16-positive cells (brown staining) relative to total number of endometrial cells (blue staining + brown staining), considering both cytoplasmic and nuclear staining as positive. The percentage of stained cells was used to determine the expression level of the p16 protein, with a higher positive rate indicating higher protein expression. Additionally, individual patient data for p16-positive endometrial cell rates and pregnancy outcomes were plotted using scatter plots to visualize the relationship between endometrial senescence markers and clinical outcomes. Visualizations were created using the ggplot2 package (version 3.5.2) in R. Given the exploratory nature of this small sample cohort, we performed only descriptive statistical analyses, calculating the median of p16-positive cells rates and clinical pregnancy rates for both the NOR and DOR groups.

### 2.5 Statistical analysis

All data analyses were performed using R software (v 4.3.2). Statistical significance was set at P < 0.05.

#### 2.5.1 Descriptive analysis

The baseline characteristics, including age, body mass index (BMI), antral follicle count, AMH, controlled ovarian stimulation protocol, total gonadotropin (Gn) dose, Gn days, number of mature oocyte (MII), fertilization methods, number of two pronuclei (2PN), cleavage embryo, endometrial preparation protocol for FET, endometrial thickness, endometrial type, number of embryos transferred, embryo stage, and embryo grade were analyzed descriptively and compared between groups using the dplyr package (version 1.1.4) and broom package (version 1.0.5) in R. Continuous variables are expressed as mean ± standard deviation (mean ± SD) if normally distributed and compared using an independent samples t-test, or as median (interquartile range) if non-normally distributed and compared using the Mann–Whitney U test. Categorical variables are expressed as proportions or percentages and were analyzed using the chi-square test. Statistical significance was set at P < 0.05.

#### 2.5.2 Inverse probability weighting and comparison

To control the impact of confounding factors when comparing pregnancy outcomes between the DOR and NOR groups, we used the IPW method (19). Statistical analyses were performed with the dplyr package (version 1.1.4), the tableone package (version 0.13.2) and the survey package (version 4.4.2). First, a multivariate logistic regression model was fitted to estimate the propensity scores, with confounding factors as independent variables and DOR as the dependent variable. The selection of confounding factors was adjusted for variables with statistically significant differences between groups (P < 0.05) in the abovementioned baseline characteristics and was also based on clinical experience. The IPW weights were calculated based on the estimated propensity scores. After IPW standardization, the distribution of the measured confounding factors between the two groups was balanced to some extent, mimicking the situation of a randomized trial. The balance of confounding factors before and after weighting was assessed using standardized mean differences (SMDs), with an absolute SMD close to 0.1 indicating a good balance.

In the IPW-weighted dataset, weighted logistic regression models were used to analyze the effect of DOR on hCG positivity, clinical pregnancy rates, and biochemical pregnancy loss rates. Logistic regression models used pregnancy outcomes as dependent variables and DOR as the independent variable while adjusting for confounding factors. Odds ratio (OR), risk ratio (RR), and 95% confidence intervals (CIs) were calculated to quantify the effects of DOR on pregnancy outcomes. The statistical analyses were performed using the EValue packages (version 4.1.3) in R.

To visually present the research findings, the following statistical plots were generated: bar plots comparing pregnancy outcomes between the two groups after weighting, plots displaying the balance of confounding factors before and after IPW, and forest plots illustrating the effect of DOR on pregnancy outcomes. The visualizations were created using the ggplot2 package (version 3.5.2) in R.

## 3 Results

Overall, 1,362 participants were included in the study, with 1,226 in the NOR group and 136 in DOR group. The baseline characteristics of the two groups are summarized in **Table 1**. Significant differences were observed between the two groups in terms of age, BMI, AMH level, Gn dose, number of MII oocytes, number of 2PN embryos, number of normally cleaved embryos, Controlled Ovarian Hyperstimulation (COH) protocols, FET medication protocols, type of transferred embryos, and grade of transferred embryos (P < 0.05).

**Table 1:** Baseline characteristics and comparison between the DOR and NOR group.

Baseline characteristics	NOR group (n = 1226)	DOR group (n = 136)	P-value
Age (years)	33 [29–35]	35 [32.75–37.25]	0.000*
BMI (kg/cm^2^)	21.98 [20.03–24.34]	23.19 [20.75–25.89]	0.001 *
AMH (ng/mL)	4.08 [2.62–6.25]	0.46 [0–0.77]	0.000*
Gn (days)	10 [9–11]	10 [8–11]	0.196
Total Gn dose	1950 [1400–2700]	2650 [1837.5–3375]	0.000*
No. of MII	14 [10–19]	7 [4–12.25]	0.000*
No. of 2PN	10 [7–14]	5 [3–9]	0.000*
No. of cleavage embryo	10 [6.25–13]	5 [3–9]	0.000*
Endometrial thickness (mm)	8.8 [8.1–9.9]	8.9 [8.2–10]	0.486
No. of embryos transferred	1 [1–2]	1 [1–2]	0.327
COH protocol			0.000*
long GnRH agonist (luteal phase)	616 (50.24%)	50 (36.76%)	
long GnRH agonist (follicular phase)	87 (7.1%)	9 (6.62%)	
GnRH antagonist	501 (40.86%)	69 (50.74%)	
Super long protocol	12 (0.98%)	0	
Mild ovarian stimulation	3 (0.24%)	5 (3.68%)	
Progesterone primed ovarian stimulation	6 (0.49%)	1 (0.74%)	
Natural cycles	0	2 (1.47%)	
Others	1 (0.08%)	0	
Fertilization methods			0.071
IVF	927 (75.61%)	110 (80.88%)	
ICSI	283 (23.08%)	22 (16.18%)	
PGT	16 (1.31%)	4 (2.94%)	
FET protocol			0.002 *
Natural cycles	345 (28.14%)	54 (39.71%)	
HRT cycles	663 (54.08%)	51 (37.5%)	
OI cycles	132 (10.77%)	16 (11.76%)	
GnRH-HRT cycles	86 (7.01%)	15 (11.03%)	
Endometrial type			0.846
A	20 (1.63%)	2 (1.47%)	
B	1131 (92.25%)	124 (91.18%)	
C	75 (6.12%)	10 (7.35%)	
Transferred embryo stage			0.001 *
Cleavage embryo	70 (5.71%)	18 (13.24%)	
Blastocyst	1156 (94.29%)	118 (86.76%)	
Transferred embryo grade			0.026 *
Low-quality	270 (22.02%)	42 (30.88%)	
Good-quality	956 (77.98%)	94 (69.12%)	
Pregnancy outcome hCG positive rate	69.74%	61.03%	0.047*
Clinical pregnancy rate	58.65%	50.00%	0.065
Biochemical pregnancy loss rate	15.91%	18.07%	0.638

AMH, anti-Müllerian hormone; BMI, body mass index; COH, Controlled Ovarian Hyperstimulation; DOR, diminished ovarian reserve; FET, frozen-thawed embryo transfer; GnRH, gonadotropin-releasing hormone; HRT, hormone replacement therapy; ICSI, intracytoplasmic sperm injection; IVF, *in vitro* fertilization; NOR, normal ovarian reserve; OI, ovulation induction; PGT, preimplantation genetic testing.

* P<0.05.

**Figure 1:**
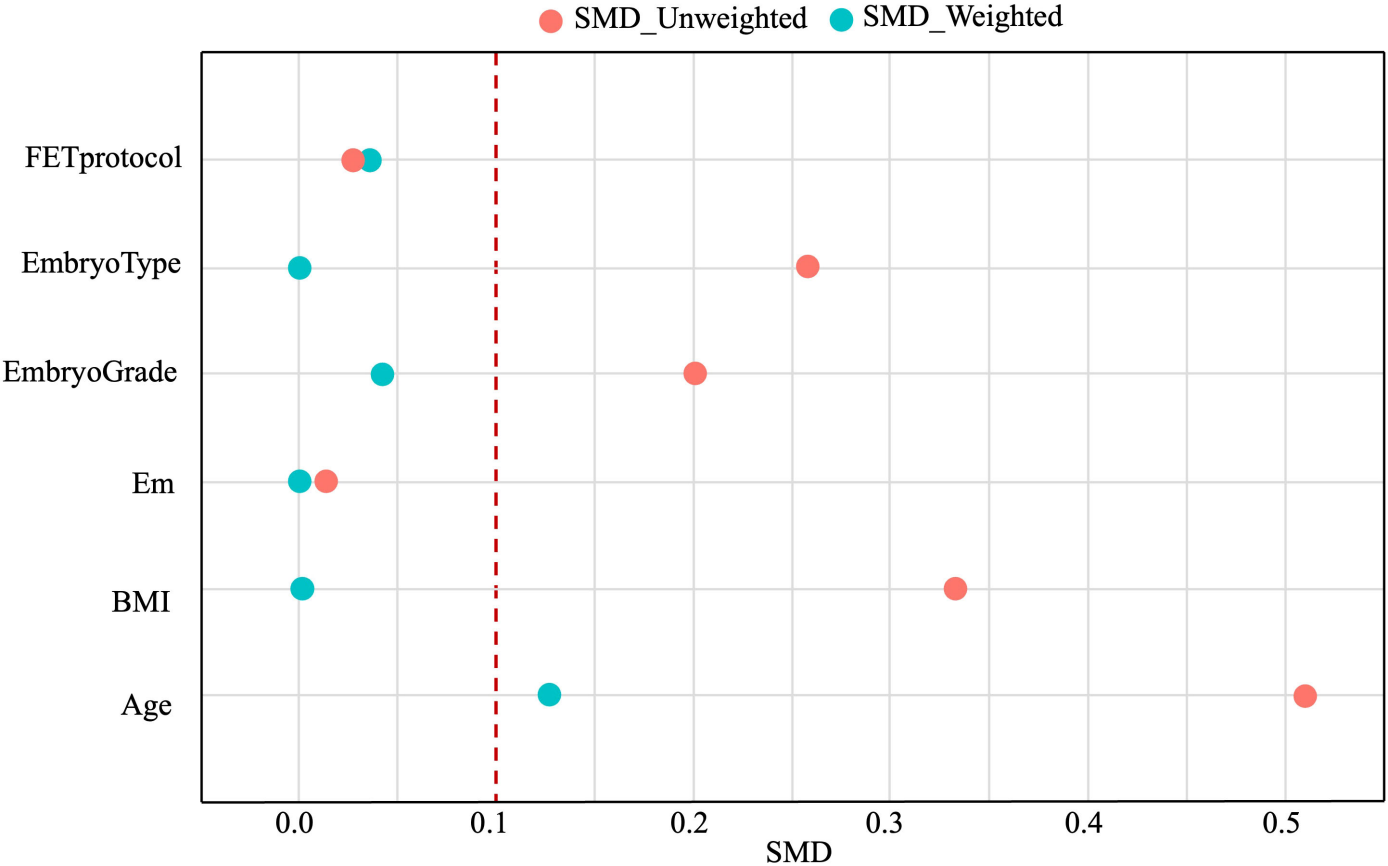
Confounding factors affecting pregnancy outcomes before (red circles) and after (blue circles) IPW. IPW, inverse probability weighting; SMD, standard mean difference.

Confounding factors, including age, BMI, endometrial thickness, FET protocols, type of transferred embryos, and grade of transferred embryos, were balanced between the two groups using the IPW method. The SMD values before and after weighting are shown in **Figure 1**. After weighting, most confounding factors achieved an SMD close to 0.1, indicating significant improvement in balance.

**Figure 2:**
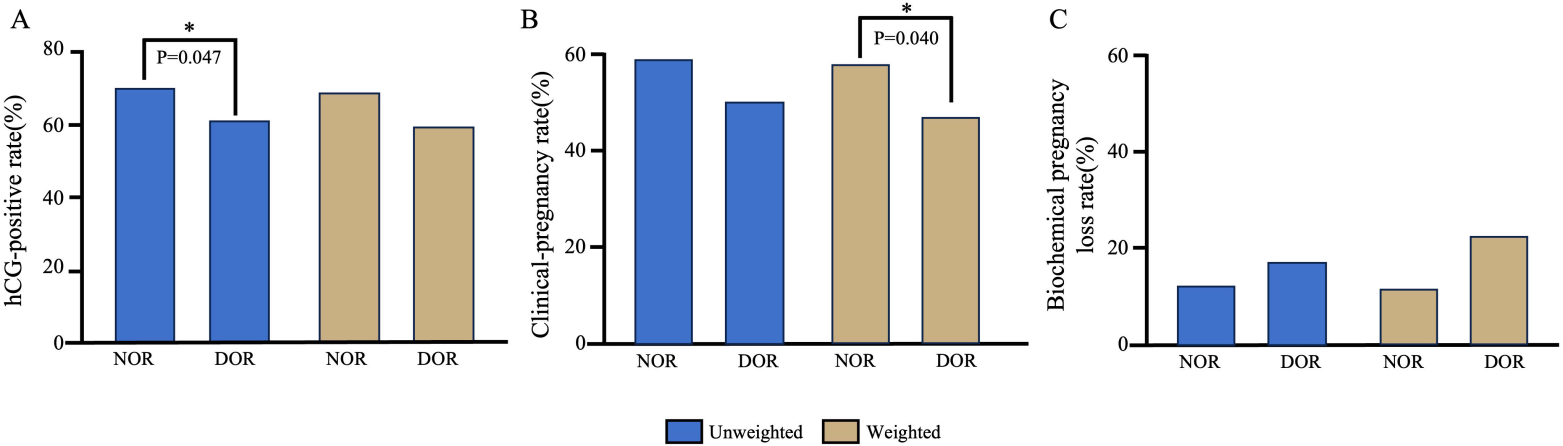
Comparison of pregnancy outcomes between the NOR and DOR groups before and after IPW. **(A)** hCG-positive rate. **(B)** Clinical-pregnancy rate. **(C)** Biochemical pregnancy loss rate. *P<0.05. IPW, inverse probability weighting; NOR, normal ovarian reserve; DOR, diminished ovarian reserve.

Pregnancy outcomes between the two groups are presented in **Table 2** and **Figure 2**. Prior to IPW, the hCG positivity rate was significantly higher in the NOR group than in the DOR group (69.74% vs. 61.03%, P = 0.047). The NOR group demonstrated higher clinical pregnancy rates (58.65% vs. 50.00%) and lower biochemical pregnancy loss rates (15.91% vs. 18.07%) than the DOR group, although these differences were not statistically significant (P = 0.065 and P = 0.638, respectively). Following IPW adjustment, no significant differences were observed in hCG positivity (69.20% vs. 60.20%, P = 0.086) and biochemical pregnancy loss rates (15.77% vs. 21.88%, P = 0.314) between the two groups. However, the clinical pregnancy rate remained significantly higher in the NOR group than in DOR group (58.30% vs. 47.00%, P = 0.040).

**Table 2:** Comparison of pregnancy outcomes between the two groups.

Pregnancy outcome	Full cohort			Inverse probability weighted cohort		
	NOR	DOR	P-value	NOR	DOR	P-value
hCG positive rate	69.74%	61.03%	0.047*	69.20%	60.20%	0.086
Clinical pregnancy rate	58.65%	50.00%	0.065	58.30%	47.00%	0.040*
Biochemical pregnancy loss rate	15.91%	18.07%	0.638	15.77%	21.88%	0.314

NOR, normal ovarian reserve; DOR, diminished ovarian reserve; hCG, human chorionic gonadotropin.

* P<0.05.

**Figure 3:**
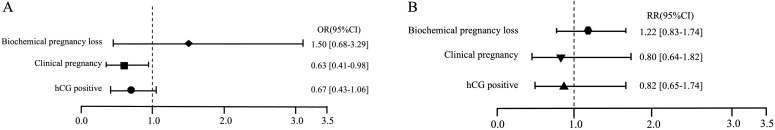
Forest plots. **(A)** Forest plots of OR comparing pregnancy outcome between the DOR and NOR groups; **(B)** Forest plots of RR comparing pregnancy outcome between the DOR and NOR groups. OR, odds ratio; RR, risk ratio; NOR, normal ovarian reserve; DOR, diminished ovarian reserve.

The weighted logistic regression model showed that for the hCG positivity rate, the OR was 0.67 (95% CI: 0.43–1.06), and the RR was 0.82 (95% CI: 0.65–1.74). For the clinical pregnancy rate, the OR was 0.63 (95% CI: 0.41–0.98), and the RR was 0.8 (95% CI: 0.64–1.82). For the biochemical pregnancy loss rate, the OR was 1.50 (95% CI: 0.68–3.29), and RR 1.22 (95% CI: 0.83–1.74). A forest plot is shown in **Figure 3**.

All 16 patients selected for IHC analysis demonstrated normal hysteroscopic findings during the proliferative phase examination. No intrauterine abnormalities, including polyps, adhesions, septum, or inflammatory changes, were observed. This confirms that the observed differences in p16 expression were not attributable to structural uterine abnormalities.

**Figure 4:**
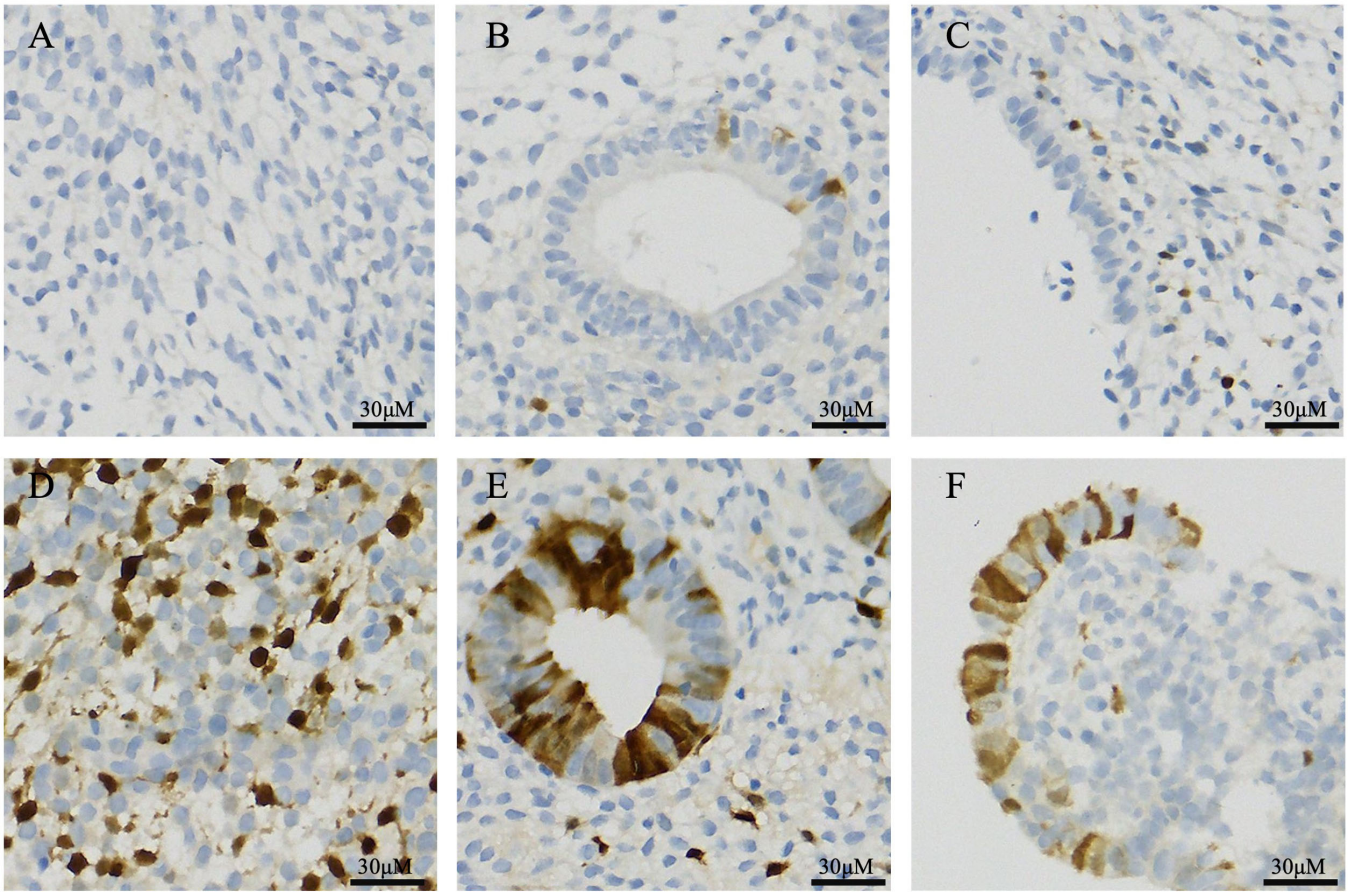
Immunohistochemical staining of p16 protein in different endometrial cell types. **(A)** Stromal cells in the NOR group; **(B)** Glandular epithelial cells in the NOR group; **(C)** Luminal epithelial cells in the NOR group; **(D)** Stromal cells in the DOR groups; **(E)** Glandular epithelial cells in the DOR groups; and **(F)** Luminal epithelial cells in the DOR groups. Brown staining indicates positive expression. Scale bars represent 30 μm. NOR, normal ovarian reserve; DOR, diminished ovarian reserve.

**Figure 5:**
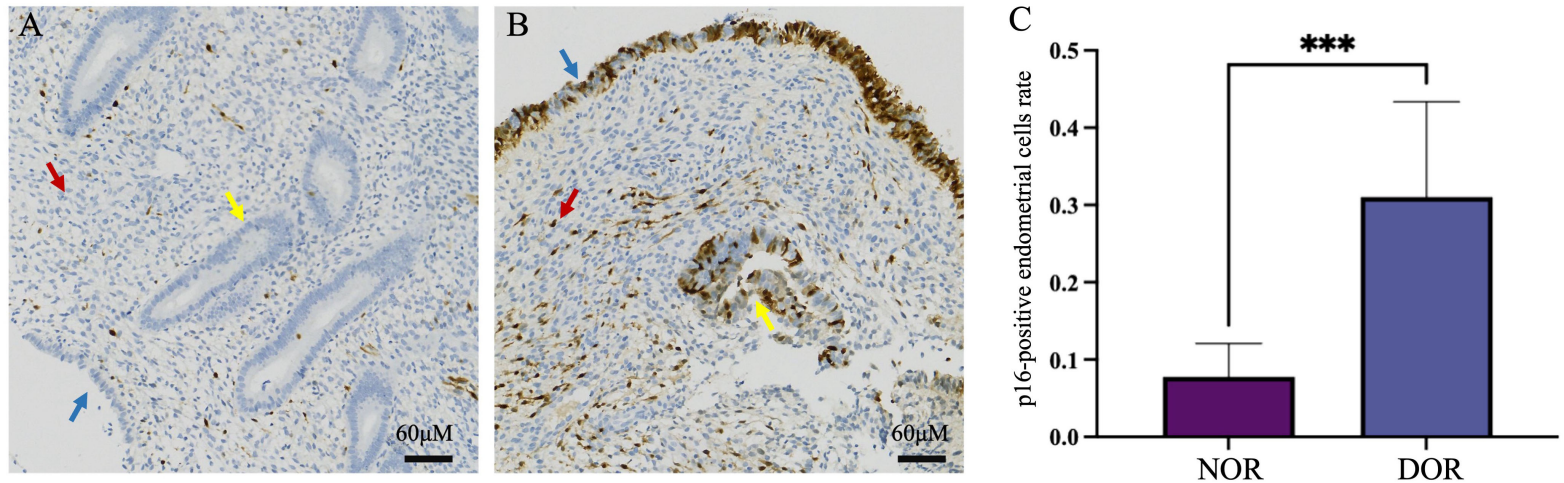
Immunohistochemical staining of p16 protein in the two groups. **(A)** NOR group; **(B)** DOR group; red arrows indicate stromal cells, yellow arrows indicate glandular epithelial cells, and blue arrows indicate luminal epithelial cells. **(C)** Analysis of immunohistochemistry. The x-axis represents the different groups, and the y-axis represents the percentage of p16-positive cells in endometrial cells. Data are presented as mean ± SD, independent experiments N = 5. ***P < 0.001 (compared with the NOR group). Scale bars represent 60 μm. NOR, normal ovarian reserve; DOR, diminished ovarian reserve; SD, standard deviation.

**Figure 6:**
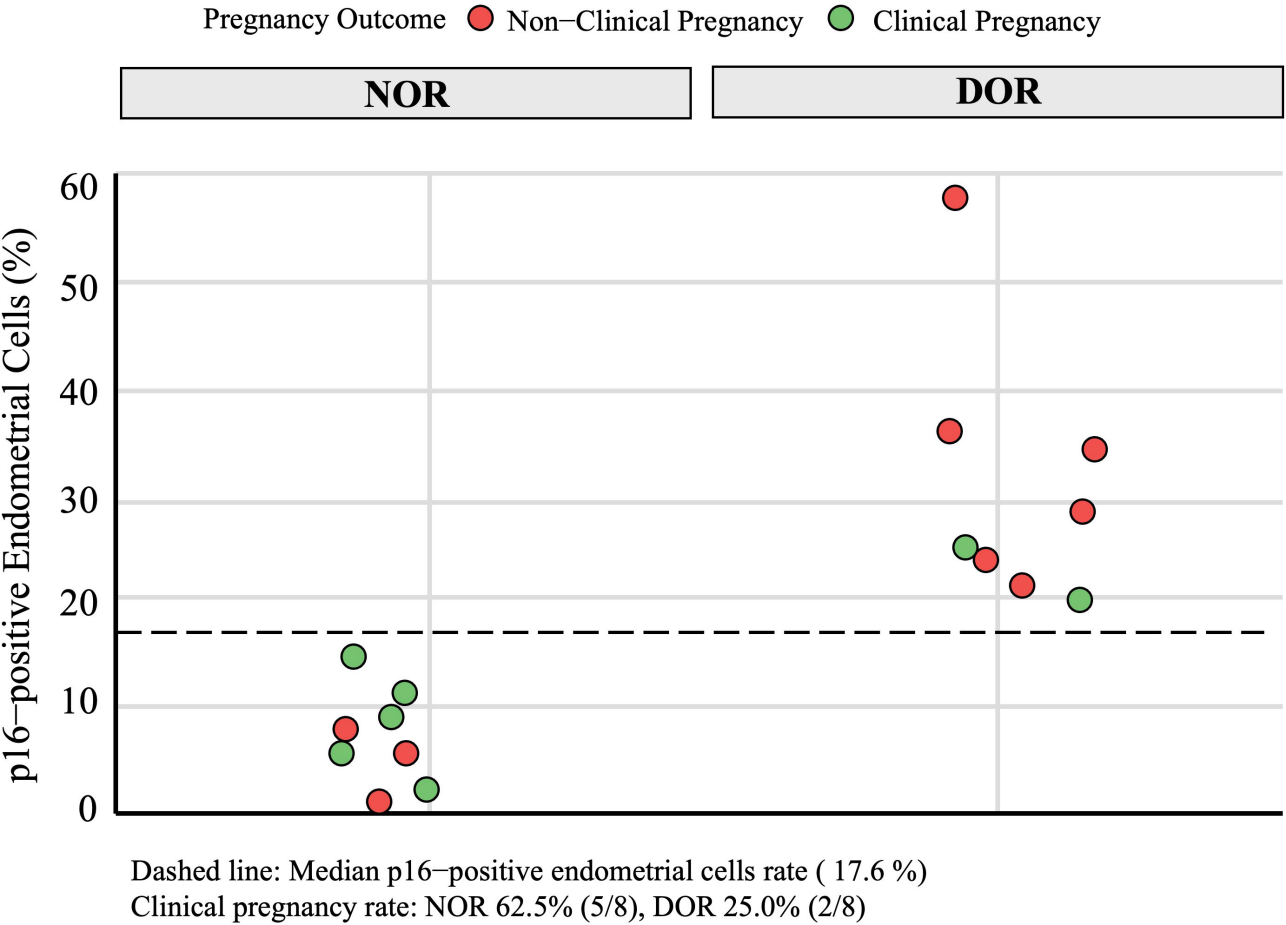
Individual patient scatter plot of p16 expression and pregnancy outcomes in immunohistochemical analysis subcohort. Green circles represent clinical pregnancy, red circles represent non-clinical pregnancy. NOR, normal ovarian reserve; DOR, diminished ovarian reserve.

In the DOR group, p16-positive staining was observed in three different types of endometrial cells: glandular epithelial cells, luminal epithelial cells, and stromal cells. Similarly, scattered p16-positive cells were also observed in the NOR group (**Figure 4**). The analysis of the positive cell rates from the IHC staining results demonstrated that the expression level of p16 protein in endometrial cells was significantly higher in the DOR group than in the NOR group (P < 0.05, **Figure 5**). The baseline characteristics of patients in the p16 expression analysis subcohort are summarized in **Supplementary Table S1**. No significant differences were observed between the two groups in terms of age, BMI, endometrial thickness, number of embryos transferred, FET protocol, stage and grade of embryos transferred (P > 0.05). Individual patient scatter plot of p16 expression and pregnancy outcomes is shown in **Figure 6**. Among the 16 patients analyzed, the median of p16-positive endometrial cells rate was 17.6%. Based on the analysis of samples from 16 patients, the clinical pregnancy rate in the NOR group, which had a low rate of p16-positive endometrial cells, was 62.5%; in contrast, the rate was 25% in the DOR group, which had a high rate of p16-positive endometrial cells.

## 4 Discussion

This study provides a comprehensive evidence demonstrating that young patients (<40 years) with infertility and DOR experienced significantly reduced clinical pregnancy rates following FET. After rigorously controlling confounding factors using IPW, we observed that the clinical pregnancy rate in the DOR group was markedly lower than that in the NOR group (47.0% vs. 58.3%, P = 0.040), with an OR of 0.63 (95% CI: 0.41–0.98). Beyond these clinical findings, our exploratory molecular analysis revealed intriguing insights into the potential underlying mechanisms. IHC examination of endometrial tissues demonstrated significantly elevated p16 expression in DOR patients, linking it to poor pregnancy outcome and suggesting that premature endometrial aging may contribute to the observed reduction in pregnancy success. These findings not only highlight the complex nature of fertility in young women with DOR but also underscore the importance of considering endometrial factors in reproductive assessments.

We first analyzed the baseline characteristics of the DOR and NOR groups and identified significant differences in variables such as age, BMI, AMH level, Gn dose, number of MII oocytes, number of 2PN embryos, and COH protocols (P < 0.05). Patients with DOR were older, had higher BMI, and exhibited significantly lower AMH levels, reflecting DOR and reduced ovarian responsiveness. These findings align with previous studies (4, 20, 21). To investigate whether reduced clinical pregnancy rates in patients with DOR could be associated with impaired endometrial receptivity rather than embryo-related factors, we employed the IPW method to adjust for confounding variables. Confounders were selected based on baseline differences between the groups, including age, BMI, endometrial thickness, FET protocols, and embryo-related factors (e.g., type and grade of transferred embryos). Variables directly related to DOR, such as AMH levels and ovarian responsiveness, were excluded to focus on factors independent of the ovarian reserve. After weighting, the SMD of these variables approached 0.1, indicating an effective balance between groups. After adjustment, the difference in hCG positivity rates was not significant (60.2% in the DOR group vs. 69.2% in the NOR group; P = 0.086); however, the clinical pregnancy rate was significantly lower in the DOR group (58.30% vs 47.00%, P < 0.05). Moreover, the biochemical pregnancy loss rate showed a notable finding, showing a higher rate of 21.88% in DOR group compared with 15.77% in the NOR group, although this difference was not statistically significant (P = 0.314). This suggests that DOR patients not only have reduced clinical pregnancy rates but also may experience a higher risk of early pregnancy loss, highlighting the potential impact of diminished ovarian reserve on early pregnancy maintenance.

The OR for clinical pregnancy was 0.63 (95% CI: 0.41–0.98), whereas the RR was 0.80 (95% CI: 0.64–1.82). The OR of 0.63 indicated that the odds of clinical pregnancy were 37% lower in DOR patients, whereas the RR of 0.80 represented a 20% relative reduction in clinical pregnancy risk for DOR patients. The 95% CI for the OR did not include 1, indicating a significant difference, whereas the 95% CI for the RR included 1, suggesting a nonsignificant difference between the two groups. Notably, the OR compared the ratio of odds of pregnancy between groups, whereas the RR compared the ratio of probabilities; because clinical pregnancy is a common outcome (control group risk >10%), the OR inherently amplified the effect size relative to the RR. Although the RR did not reach statistical significance, both its point estimate (0.8) and that of the OR (0.63) consistently confirmed DOR as an independent risk factor for reduced clinical pregnancy rates. This finding emphasized that DOR is more than an ovarian factor—it includes implications for uterine receptivity. However, the RR value suggested that DOR’s effect on achieving pregnancy is moderate, warranting further exploration of additional modifying factors.

The reduced pregnancy rates in young DOR patients likely result from multiple interconnected mechanisms beyond ovarian factors alone. The essence of DOR lies in the aging tendency of the ovaries, which may share similar pathological changes across multiple organs, a characteristic of age-related aging. Studies have shown that the miRNA profile in patients with DOR resembles that in older women (22), accompanied by reduced estrogen secretion, elevated basal follicle-stimulating hormone levels, and alterations in the ovarian microenvironment. These pathological changes impact ovarian function and may trigger premature endometrial aging, potentially compromising endometrial receptivity and embryo implantation potential (23, 24).

Endometrial aging stems from the senescence of endometrial cells, which is characterized by irreversible cell cycle arrest in both proliferative and postmitotic cells (24). Marked by increased p16 expression, endometrial aging alters cellular senescence balance, leading to the accumulation of senescent stromal cells and excessive secretion of SASP. This prolonged pro-inflammatory environment disrupts critical pathways involved in decidualization, endometrial stromal cell organization, and hormonal responsiveness, consequently impairing endometrial receptivity and reducing the likelihood of successful embryo implantation (25, 26). Research has shown that human endometrial stromal cells from nonpregnant patients exhibit higher proportions of senescent cells and increased p16 expression during the proliferative phase, potentially hindering implantation (27). Elevated p16 levels have also been documented in the luminal and glandular epithelial cells in older women, correlating with reduced receptivity in women aged >45 years (28, 29). These findings suggested the potential association between endometrial aging and decreased implantation potential in patients with DOR.

In the IHC analysis subcohort, we observed intriguing variations in pregnancy outcomes and p16 expression between the NOR and DOR group: a significantly higher p16 expression was observed in the DOR group (P < 0.05), with p16-positive staining localized in both luminal and glandular epithelial cells. Moreover, a trend toward lower clinical pregnancy rates was observed with higher p16 expression, although this association should be interpreted with caution due to the small sample size and potential confounding factors. The small sample size (n = 16) for p16 expression analysis and the age distribution trend toward older ages in the DOR group (despite no statistically significant difference) may limit the robustness of this association. Notably, the pregnancy rates markedly differed in the subcohort: patients in the NOR group demonstrated a 62.5% clinical pregnancy rate, which generally aligned with the large-cohort analysis, whereas patients in the DOR group showed a significantly lower rate at 25%. This discrepancy might be attributed to potential sampling bias in the small subcohort. Critically, elevated p16 expression in DOR patients suggests premature endometrial aging, suggesting population heterogeneity wherein subgroups with pronounced cellular senescence exhibit poorer reproductive outcomes. These preliminary observations raise the possibility that cellular senescence may contribute to divergent reproductive outcomes, with markers like p16 potentially enabling high-risk subgroup identification. While these findings provide a valuable framework for future research, further validation in larger, well-controlled studies is necessary to address the limitations of the small sample size and age distribution bias. Such studies will help establish the mechanistic relationships between p16 expression, endometrial aging, and clinical pregnancy outcomes.

This study has some limitations that warrant careful consideration when interpreting the results. A critical limitation is the absence of systematic preimplantation genetic testing (PGT). While we employed IPW to adjust for observed differences in age, embryo grade, and embryo stage between groups, the lack of PGT does not allow completely rule out age-related embryo aneuploidy as a contributing factor to the observed pregnancy rate differences. This limits our ability to distinguish the independent effects of endometrial aging from those of age-related chromosomal abnormalities. Additionally, the substantial difference in embryo availability and quality between groups is a major confounding factor. The total number of cryopreserved embryos, along with embryos type and grade, inherently impacts the probability of selecting high-quality embryos for transfer, potentially confounding the relationship between DOR and pregnancy outcomes. While IPW adjustment balanced most measured confounders, several methodological concerns remain: (i) the inability to account for embryo cohort size and quality distribution at the time of FET, which more accurately reflects actual embryo selection opportunities; (ii) residual confounding from unmeasured age-related factors that cannot be captured through observational data; and (iii) the biological reality that statistical adjustment cannot eliminate the inherent increase in aneuploidy rates with advancing maternal age.

Another limitation is that the immunohistochemical analysis was conducted on a relatively small sample (n = 16), which limits the generalizability of the p16 expression findings and statistical power for detecting smaller effect sizes. Our assessment of endometrial aging relied solely on p16 expression. A multimarker approach incorporating additional senescence markers and functional assessments would provide more comprehensive evidence of endometrial aging. While we observed elevated p16 expression occurring alongside reduced pregnancy rates, owing to the study design, we could not establish causality or elucidate the underlying mechanisms linking endometrial senescence to implantation failure. However, these preliminary findings align with the hypothesis that elevated p16 expression and premature endometrial aging may be involved in impaired endometrial receptivity in DOR, warranting mechanistic validation.

Future research should focus on large-sample prospective clinical studies that utilize PGT to eliminate the impact of aneuploid embryos, allowing for a concentrated analysis of differences in pregnancy outcomes based on embryonic implantation. Increasing the sample size will facilitate the assessment of multiple cellular aging markers and endometrial receptivity indicators in relation to endometrial aging across two groups. Furthermore, future research should explore the correlations between these factors and their clinical implications for pregnancy outcomes, drawing a comprehensive evidence chain connecting DOR, endometrial aging, decreased receptivity, and adverse pregnancy outcomes.

## 5 Conclusion

This preliminary study provides valuable insights into the multifactorial nature of reproductive failure in DOR patients and establishes a foundation for future mechanistic investigations. We found that young women (<40 years old) with DOR exhibited reduced pregnancy rates. Exploratory molecular analysis suggested the potential involvement of endometrial aging pathways, as evidenced by elevated p16 expression. These findings provide exploratory insights into the potential role of endometrial factors in reproductive outcomes of young DOR patients. However, due to potential confounding factors and exploratory nature of this study, these results should be interpreted with caution. Future large-scale prospective studies are warranted to validate these observations and elucidate causal mechanisms.

## Data Availability

The raw data supporting the conclusions of this article will be made available by the authors, without undue reservation.
