# Supplementary material for: Clinical pregnancy outcomes in young women with diminished ovarian reserve undergoing frozen embryo transfer: a comprehensive analysis with exploratory insights into endometrial aging
Source: Front Endocrinol (Lausanne). 2025 Jul 29;16:1608200. doi: 10.3389/fendo.2025.1608200 (PMC12339334; doi:10.3389/fendo.2025.1608200)
Supplement: Supplementary file 1 [file Table1.docx]

Supplementary Material

**Table S1. Baseline characteristics of patients in p16 expression analysis subcohort**

| Baseline characteristics | NOR (n = 8) | DOR (n = 8) | P-value |
| --- | --- | --- | --- |
| Age (years) | 32.88±4.09 | 36.12±2.75 | 0.086 |
| BMI (kg/cm^2^) | 21.06±2.52 | 22.03±2.92 | 0.489 |
| Endometrial thickness (mm) | 9.38±2.13 | 8.89±1.28 | 0.589 |
| No. of embryos transferred | 1 [1–2] | 2 [1.75–2] | 0.161 |
| FET protocol |  |  | 0.287 |
| Natural cycles | 5 (62.5%) | 3 (37.5%) |  |
| HRT cycles | 3 (37.5%) | 3 (37.5%) |  |
| GnRH-HRT cycles | 0 (0) | 2 (25%) |  |
| Transferred embryo stage |  |  | 1 |
| Cleavage embryo | 1 (12.5%) | 1 (12.5%) |  |
| Blastocyst | 7 (87.5%) | 7 (87.5%) |  |
| Transferred embryo grade |  |  | 0.313 |
| Low-quality | 2 (25%) | 5 (62.5%) |  |
| Good-quality | 6 (75%) | 3 (37.5%) |  |

BMI, body mass index; FET, frozen-thawed embryo transfer; DOR, diminished ovarian reserve; GnRH, gonadotropin-releasing hormone; HRT, hormone replacement therapy; NOR, normal ovarian reserve
